# One-Finger Gripper for Microobjects to Submillimeter-Sized Objects Based on Temperatures of Dew and Freezing Points

**DOI:** 10.3390/mi17050573

**Published:** 2026-05-05

**Authors:** Božidar Bratina, Dušan Fister, Jernej Nezman, Jakob Šafarič, Riko Šafarič

**Affiliations:** 1 Faculty of Electrical Engineering and Computer Science, University of Maribor, 2000 Maribor, Slovenia; bozidar.bratina@um.si (B.B.); jernej.nezman@student.um.si (J.N.); riko.safaric@um.si (R.Š.); 2 EMSISO d.o.o, Pesnica pri Mariboru 20A, 2211 Pesnica pri Mariboru, Slovenia; jakob.safa@gmail.com

**Keywords:** one-finger gripper, microobjects, submillimeter-sized objects, capillary force, van der Waals force, coupling force due to ice

## Abstract

The new method proposed in this study, featuring a one-finger gripper, uses three types of forces—van der Waals force, capillary force, and coupling force due to ice—to grip and release microobjects to submillimeter-sized objects (5 to 300 µm). The gravitational force of an object can be neglected in the case of microobjects, but this is not the case for submillimeter-sized objects. This is the first reason that we use the coupling force due to ice; the second reason is that the shape of a micro- or submillimeter-sized object does not matter in this case. The usage of all three forces yields greater versatility regarding objects of different sizes and shapes and, consequently, greater overall reliability in gripping or releasing compared with methods that use only one or two of the mentioned forces. In this study, the laboratory set-up involved the active control of the temperature for both the one-finger gripper and the releasing surface for objects from −25 °C to 40 °C in a closed dust-free chamber in atmospheric air at relative humidity (RH) = 30%. A relatively low RH was achieved with the RH controller, enabling the release or grip procedures to last approx. 2–3 s for microobjects and 6 s for submillimeter-sized objects with the same equipment.

## 1. Introduction

Microassembly, or nanoassembly, is an emerging technology in which micro- and nanoobjects are used to build 3D structures. Due to the complex operations involved, micro- and nanoassemblies require the following abilities:

- Pick-up (grip) individual micro- or nanoparticles;
- Move particles in a 3D space;
- Position them with nanometer precision;
- Release them actively in a dedicated place so that they can bond, fuse, or assemble with other particles.

Optical tweezers have all of these abilities and are considered the most well-known tool for nano- and microassembling. The father of optical tweezers won the Nobel prize in 2018, while his main research work was published between 1970 [1] and 1986 [2]. Optical tweezers are universally used to manipulate (1) transparent dielectric nanoparticles in transparent liquids, (2) highly reflective metallic objects, and (3) highly optically absorbable objects [3]. Recent developments have included laser beams for the rotation of an ellipsoid rotor in a liquid medium [4]. Optical tweezers can be used in liquids, gases, and even in a vacuum, with a single limitation in practice, i.e., their minimal gripping (dragging) force, which is in the pN range. Such a small range makes them suitable only for objects of minimal size, <50 µm consecutively, since, for larger objects, the gravitational force becomes dominant and overcomes the gripping force of the optical tweezers, making them ineffective for gripping. Theoretically, optical tweezers can handle objects larger than 50 µm, but only with underlying strong laser beams that can destroy the objects thermally. Also, gripping the object from the surface has also been proven to be problematic, due to the presence of a van der Waals force (vdW) that acts between the object and surface. Specifically, a vdW is a few hundred times larger than the gripping force of optical tweezers for objects with dimensions greater than 1 µm, and this remains an unresolved challenge.

In recent decades, gripping techniques have been developed to address this challenge. Various grippers have been developed up to now, including a multi-finger microgripper, two-finger microgrippers, a one-finger submerged freezing microgripper, an air freezing one-finger microgripper, a vdW-based one-finger micro/nanogripper, and a capillary-based one-finger microgripper. All of these techniques enable microobjects to submillimeter-sized objects to be gripped from the surface successfully, but some of them struggle with releasing the objects back onto the surface, as well as with using them to build microstructures.

The multi-finger microgripper [5] and two-finger microgripper [5,6,7] cannot release objects of sizes <100 µm reliably, due to sticking on one of the fingers during release (which finger the object sticks to is always unknown). Consequently, the release of the object is inaccurate or impossible. The second reason is that the finger–object vdW is similar to the object–surface vdW. Thus, a multi-finger microgripper is only reliable for larger objects in the submillimeter range, where gravity is strong enough to pull the objects to the platform. Different experiments have been implemented to show this. For example, the authors of [8] and [5] worked with typical object sizes of 400 µm and object weights of 0.25 mg, while [6] worked with objects of sizes between 100 and 300 µm. The authors of [7] worked with the smallest objects of dimensions 400–500 µm.

Most microgrippers are two-fingered, with rare implementations using one finger. Microgrippers with one finger tend to rely on a capillary force [9,10,11,12,13,14,15] for grip operations and on vdW for release operations. Typically, an object is released by drying out the water droplet between the finger and object, breaking the capillary force [9,10,11,15]. Consequently, the remaining force between the tip of the one-finger gripper and object is vdW, which is typically 5–10 times smaller than capillary force. Hence, grippers relying on capillary force are particularly difficult to use for release operations. The experiments described in [9,10,11,12] used gravitational force to release the object from the tip of the finger, implying that the objects must have dimensions larger than 100 µm. The research paper [13] and a book chapter [14] described the theoretical background for the gripping and release of microobjects using capillary force. The experiment described in [15] used capillary force to release an object. A small droplet was produced with a nozzle on the releasing surface, and then the bottom part of the object was placed in the droplet, where a capillary force was created between the object and surface, which was several times greater than the vdW of dry contact between the object and tip of the one-finger gripper. In this experiment, the size of the object was approx. $1\;{\mathrm{mm}}^{3}$, since the droplet was relatively large due to the surface tension of the water droplet. The experiment in [16] used microobjects with drastically reduced dimensions, 20–100 µm. They used a classical capillary force gripping method, in which the droplet was propelled by the nozzle, but an inertial method (a jolt of force) performed the release.

Next, a condensation mechanism based on air moisture [17] was exploited to produce a small amount of water, which allowed the existence of capillary force. The golden tip of the gripper’s finger was cooled slightly below the temperature of the dew point, so a thin layer of water condensed on the finger. The layer was so thin that the smallest objects pressed on the tip of the finger had dimensions of 5 µm and the largest spherical objects were approx. 50 µm in size. Larger objects required the condensation of more water on the tip to produce sufficient gripping force. At the same time, the surface had to be warmed slightly more than the temperature of the dew point, so that the condensed water on the surface evaporated and only vdW existed between the surface and object. The opposite is required when the object is released from the tip of the one-finger gripper. The contact between the object and tip of the finger is dry, due to the temperature of the tip being above the dew point, but the temperature of the surface is below the dew point; thus, capillary force is created between the object and surface. The next group of one-finger grippers are called submerged freezing one-finger microgrippers, which grip, move, and release microobjects in water [18,19,20]. They freeze the tip of the one-finger gripper, so an ice ball forms around the tip. The object in the water is frozen to the ice ball. When the object is released, the tip of the one-finger gripper is heated above the freezing temperature, melting the ice ball and releasing the object via gravity [18,20]. Of course, such a method is useful for an object with dimensions greater than 100 µm. The experiment presented in [19] used vdW in medium water to release the object on the surface and proved to be useful for an object with dimensions below 100 µm.

The freezing of the ice ball on the tip of the one-finger microgripper is also possible in the air, implemented as an air-freezing one-finger microgripper [21,22,23,24]. The experiment presented in [21] used a one-finger gripper, which was cooled with liquid nitrogen, to produce an ice ball on the tip from the droplet of water applied to the tip. During the release, the ice ball is warmed and melted with warm-air venting. To prevent the creation of capillary force between the object and tip, the object is dried via venting (evaporating the water) and then released due to gravity. This method is suitable for microobjects larger than 100 µm and submillimeter objects. Next, the experiment in [22] shows a single-finger gripper that binds moisture from the air at the tip of the gripper and freezes the object. A ball of ice forms at the tip, which prevents the precise placement of the object. The experiment only demonstrates the gripping of objects larger than 500 µm, which allows for the gravitational placement of the object.

The last type of one-finger gripper discussed here is a variant of an ice gripper, i.e., the vdW-based one-finger microgripper, or nanogripper, which operates in a pressure chamber with an ambient air pressure below p< 100 µbar, saturated with water vapor [23,24]. Such a low pressure enables the formation of an ice ball on the one-finger gripper from the moisture in the water-saturated air inside the chamber via direct deposition when the tip temperature is below −42 °C. The sublimation of the ice ball occurs at a temperature slightly above −42 °C. This deposition/sublimation effect is possible because both the temperature and pressure are below the triple point in the temperature–pressure diagram of water [25]. The sublimation/deposition process prevents parasitic capillary forces between the tip and object during release, as well as between the surface and object during gripping. The method is suitable for nano/microobjects with dimensions between 800 nm and 50 µm. Table 1 provides an overview of the most important characteristics of the microgrippers mentioned above.

The novelties of the one-finger gripper presented in this paper are as follows:

- Interaction with three forces: The proposed one-finger gripper is a single piece of equipment that can interact with three forces, i.e., vdW, capillary force, and coupling force due to ice, apart from the gravitational force.
- Greater versatility in terms of the shape and size of objects and, consequently, better reliability of gripping and releasing procedures due to interactions with the three forces (instead of the two forces listed in Table 1, which have even been tested separately for submillimeter-sized objects, 100–1000 µm, and microobjects, 1–100 µm).
- Insensibility of the shape and surface variability of the microobjects that are being gripped or released in dimensions from 5 µm to 300 µm, due to the ability of gripping objects by coupling force due to ice (again, the methods described in Table 1 were tested for spherical objects only).

The abovementioned three forces (for gripping or releasing) can be achieved using the same gripper equipment through a simple change in temperatures on the tip of the gripper or on the plane surface. However, the ability to interact with the three forces comes with the need for humidity control, which can be implemented efficiently on such a small scale using elementary actuators.

**Table 1:** Overview of the gripping mechanisms and object dimensions. Remark: *—estimation made by the authors of this paper. **—coupling force due to ice.

Refs.	Type	Medium	Gripping Force	Releasing Force	Object Dimensions
[8]	Multi-finger	Air/vacuum/water	Mechanical coupling	Gravity	100–1000 µm *
[5,6,7]	Two-finger	Air/vacuum/water	Mechanical coupling	Gravity	100–1000 µm *
[9,10,11,12]	One-finger	Air	Capillary	Gravity	100–1000 µm *
[15]	One-finger	Air	Capillary	Capillary	
[16]	One-finger	Air	Capillary	Inertial	20–100 µm
[17]	One-finger	Air	Capillary	Capillary	100–1000 µm *
[18,20]	One-finger	Water	Ice coupling **	Gravity	100–1000 µm *
[18]	One-finger	Water	Ice coupling **	Van der Waals	10–100 µm *
[21,22]	One-finger	Air	Ice coupling **	Gravity	100–1000 µm *

## 2. Materials and Methods

In this study, force measurements depend on the quality of the material and adequate laboratory preparations. Therefore, before conducting any measurements, the surface quality of the materials was tested to determine the contact distances between the microobjects in contact [26].

### 2.1. Materials

Polystyrene spheres (diameter 30 µm) were purchased from Kisker Biotech GmbH & Co. KG, Steinfurt, Germany. SiO_2_ (amorphous) spheres with diameters of 2–10 µm, 10–30 µm, and 50–100 µm were obtained from PolySciences, Inc., Warrington, PA, USA. The one-finger gripper was made of golden wire (Premion 99.995%), with a diameter of 50 µm, purchased from Alfa Aeser GmbH & Co. KG, Germany, now Thermo Fisher Scientific Inc., Waltham, MA, USA. The tip of the one-finger gripper was made of a polystyrene sphere with a diameter of 30 µm glued to the tip of golden wire with hydrophobic epoxy, purchased from Bondic GmbH, Munich, Germany. An aluminum thermal conductive paste Prolimatech PK-3, with a conductivity of $11.2\;W/m^{2}$, was purchased from Mlacom d.o.o., Ljubljana, Slovenia. It was used to glue the golden one-finger gripper to the Peltier of the 3D nanoprecision robot system. The indicating silica gel, with a grain size of 2–5 mm, purchased from Isolatech e.K., Langenfeld, Germany, was used as a desiccant for the relative humidity (RH) control system.

### 2.2. Laboratory Set-Up

**Figure 1:**
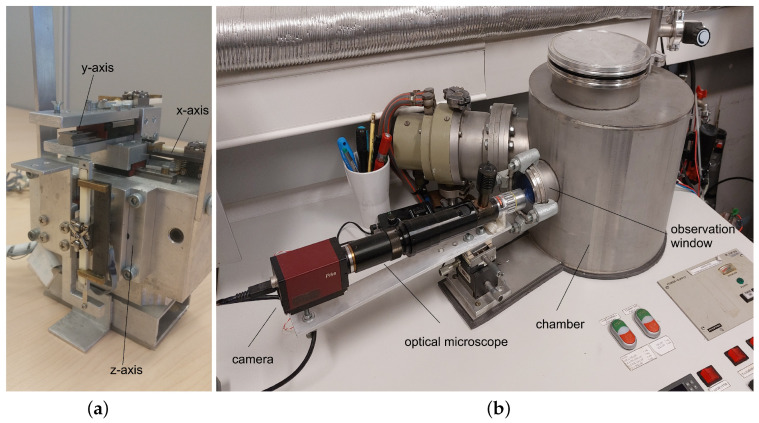
Left (**a**): 3D nanoprecision robot mechanism. Right (**b**): Dust-free chamber with support devices.

A 3D (Cartesian type of mechanism) nanoprecision robotic system [23,24,25,27], with connected *x* and *y* axes and a separated *z*-axis (see Figure 1a), was used to move the one-finger microgripper with precision in a closed loop ±61 nm with a magnetic precision incremental encoder, or accuracy in an open loop ±3.9 nm without the incremental encoder. The nanoprecision robot mechanism was installed in a dust-free chamber with a controlled RH atmosphere and a small observation window, where the robot’s movements were observed through an optical microscope and a camera (see Figure 1b).

**Figure 2:**
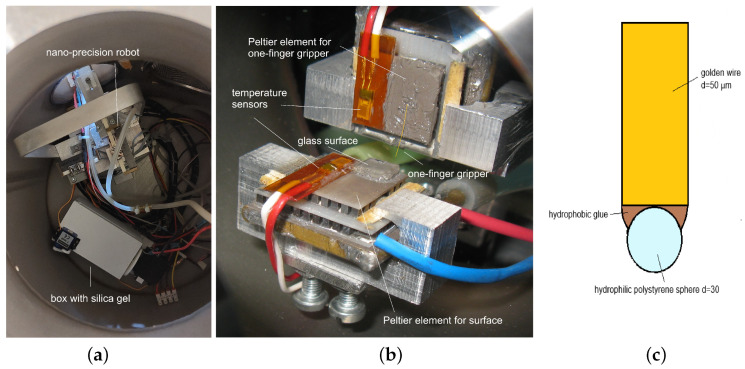
Left (**a**): Inside the chamber. Middle (**b**): Cooling/warming devices used with the one-finger gripper. Right (**c**): Sketch of the one-finger gripper.

The chamber contains the robot mechanism and a box with silica gel desiccant, which is used as an actuator for atmosphere control by RH (see Figure 2a). The Peltier element used to cool and warm the one-finger gripper was mounted on the tip of the *y*-axis, while the one-finger gripper was glued to the Peltier element with conductive paste and directed towards the *z*-axis (see Figure 2b). Another Peltier element was mounted to cool and heat the glass surface at the tip of the *z*-axis. The glass surface was fixated to the Peltier element with conductive paste. Temperature sensors were used to control the temperature of both Peltier elements. The tip of the one-finger gripper was made of a polystyrene sphere with a diameter of 30 µm, glued to the tip of the gold wire (*d* = 50 µm) with hydrophobic epoxy (see Figure 2c).

Precise control over the humidity in the chamber is required to maintain the water and ice formation dynamics under control. Hence, this research work paid special attention to the development and testing of the humidity control methods and controller.

#### Relative Humidity Control in the Chamber

**Figure 3:**
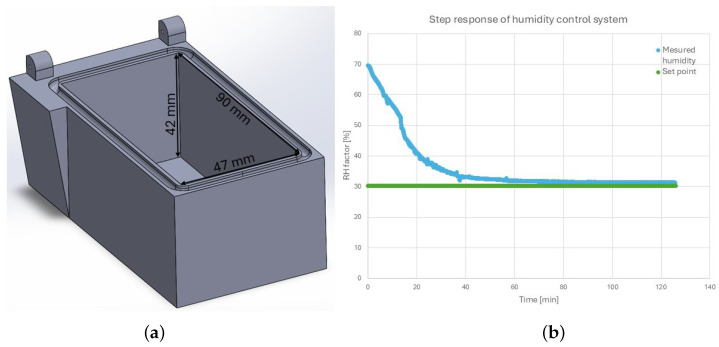
Left (**a**): Internal compartment of the silica gel box with dimensions. Right (**b**): Step response of the RH control system.

The silica gel box (the RH actuator) made of polyethylene terephthalate glycol has a rectangular internal compartment (see Figure 3a) filed with desiccant and a hinged lid that rotates with a small servomotor attached to it (see Figure 2a). By opening the box, more air can access the internal compartment, increasing the rate of desiccation, thus enabling control of the RH within the chamber. Before each use, the silica gel was prepared by drying it in a conventional convection oven at 120 °C for 3 h and allowing it to cool to room temperature. The internal compartment was filled, and the box was placed inside the chamber, where the RH was increased with an external humidifier to RH = 70%. A PI controller was then used to reach the desired set point with a static error of 1.4% after 80 min (see Figure 3b). One filling of the box with desiccant was enough for 72 h of non-stop work with the occasional opening of an upper aperture of the dust-free chamber. The RH of the air outside the lab chamber varied from RH = 70% in summer to RH = 30% in the winter.

## 3. Theoretical Background of Acting Forces

The forces used for the microobject manipulations described in this paper are the capillary force, vdW, and coupling force due to ice, and their combinations. First, theoretical models of the forces are presented, followed by methods and applications of their use for microobject manipulation, and, finally, the theoretical values are compared to the experimental measurements. The force interactions are also presented and validated between the sphere–sphere and sphere–plane combinations.

### 3.1. Capillary Force

**Figure 4:**
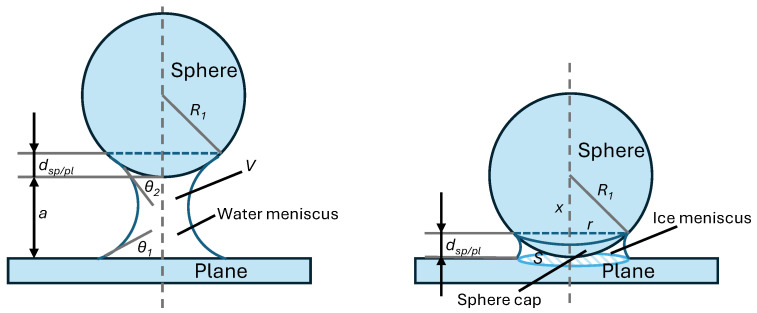
Calculation of the capillary force (**left**) and coupling force due to ice (van der Waals), with ice instead of a vacuum between the two surfaces (**right**). The relative distances in the figure are not accurate, and are for illustrative purposes only.

The theoretical capillary force between two spheres is determined by Equation (1), taking into account the calculations of dimensions, displacements, and wetting angles (see Equations (1) and (2)). Similarly, the theoretical capillary force between a sphere and a plane is presented in Equations (3) and (4). For the latter, Figure 4 (left) depicts a water meniscus formation between a sphere and a plane. All four equations for the calculation of capillary force use Rabinovich’s model and Lambert’s correction [28,29].

(1) $$F_{cap-sp/sp}=-\frac{2\cdot \pi \cdot R\cdot \gamma \cdot \cos\;\theta }{1+(a/\left(2\cdot d_{sp/sp}\right))},$$

(2) $$R=\frac{2\cdot R_{1}\cdot R_{2}}{R_{1}+R_{2}},\;\;\;d_{sp/sp}=\frac{a}{2}\cdot \left[-1+\sqrt{1+\frac{2\cdot V}{\pi \cdot R\cdot a^{2}}}\right],\;\;\;\cos\left(\theta \right)=\frac{\cos\;\theta_{1}+\cos\;\theta_{2}}{2},$$

where

(3) $$F_{cap-sp/pl}=-\frac{4\cdot \pi \cdot R_{1}\cdot \gamma \cdot \cos\;\theta }{1+(a/d_{sp/pl})},$$

where

(4) $$d_{sp/pl}=a\cdot \left[-1+\sqrt{1+\frac{V}{\pi \cdot R_{1}\cdot a^{2}}}\right].$$

Here, $R_{1}$ and $R_{2}$ are the radii of the spheres; a = 1.2 µm is the distance between the spheres, or a = 0.5 µm for the distance between the plane and sphere; γ=73 mN/m is the surface tension of the water; and $V_{sp/pl}=0.2\cdot 10^{-15}\;m^{3}$ and $V_{sp/sp}=0.235\cdot 10^{-15}\;m^{3}$ are the estimated volumes of the water bridge (meniscus) between the sphere and a plane and between two spheres, respectively. The volume of the water bridge is larger between two spheres than between a sphere and a plane, as some of the water is added to the lower sphere. $\theta_{1}=25^{\circ }$ and $\theta_{2}=25^{\circ }$ are contact angles [30].

### 3.2. Van Der Waals Force

Equation (5) was used to calculate the vdW between two spheres, and Equation (6) to calculate the vdW between a sphere and a plane [28]:

(5) $$F_{vdW-sp/sp}=-\frac{R_{1}\cdot R_{2}}{R_{1}+R_{2}}\cdot \frac{A}{6\cdot d^{2}},$$

(6) $$F_{vdW-sp/pl}=-\frac{R_{1}\cdot A}{6\cdot d^{2}},$$

Here, A=65 zJ is the Hamaker coefficient between SiO_2_ and SiO_2_ through medium air, d=0.37 nm is the separation or contact distance in the presence of nanoroughness in the microobjects [26] between a sphere and a plane, d=0.42 nm is the distance between the spheres, and $R_{1}$ and $R_{2}$ are the radii of the spheres.

### 3.3. Coupling Force Due to Ice

The theoretical model for the calculation of the coupling force due to ice was derived from the vdW equations valid for interactions between two parallel surfaces, with ice as the medium instead of air between the surfaces. The difference between the capillary force calculations is that, here, the microobjects are in contact when the water meniscus is formed and are frozen by cooling. In this manner, the medium between the microobjects presents a layer of ice instead of air (or vacuum), which greatly reduces the interaction forces. In the case of a sphere–plane interaction, as shown in Figure 4 (right), the ice meniscus between the two surfaces is defined by a surface area on the bottom of a sphere (sphere cap) and the surface of the plane. The surface area *S* of the sphere cap is calculated using Equation (7):

(7) $$S=2\cdot \pi \cdot R_{1}\cdot d_{sp/pl},$$

where $R_{1}$ is the radius of a sphere, and $d_{sp/pl}$ is the displacement of water (ice) or the height of the meniscus that covers the surface of the sphere. The surface area at the bottom of the plane can be calculated by the surface of a smaller cross-section of radius *r* defined at the height of the meniscus Equation (8):

(8) $$r=\sqrt{R_{1}^{2}-{\left(R_{1}-d_{sp/pl}\right)}^{2}},$$

and the area *S* is then $S=\pi \cdot r^{2}$. Since the curvature of the sphere cap surface in contact with the bottom plane is small, the coupling force due to ice can be calculated using the vdW formula between two parallel planes and ice between them acting as medium Equation (9):

(9) $$F_{ice-sp/pl}=-\frac{A_{SiO_{2}-ice-SiO_{2}}}{6\cdot \pi \cdot d^{3}}\cdot S,$$

where $A_{SiO_{2}-ice-SiO_{2}}$ is the effective Hamaker coefficient for glass–ice–glass interaction, *d* is the distance between the microobjects, and *S* is the surface area of the ice coupling. The effective Hamaker coefficient can be calculated using Equation (10):

(10) $$A_{SiO_{2}-ice-SiO_{2}}\approx {\left(\sqrt{A_{11}}-\sqrt{A_{33}}\right)}^{2},$$

where $A_{11}$ is a Hamaker coefficient for glass–vacuum–glass interaction $\approx 65\cdot 10^{-21}$ J, and $A_{33}$ is a Hamaker coefficient for ice–vacuum–ice interaction $\approx 60\cdot 10^{-21}$ J [31].

### 3.4. Theoretical Forces and Force Ratios Calculated

The theoretical calculations of the ratio between the capillary force and vdW for some sphere sizes vs. spheres or planes are presented in Table 2 and Table 3.

The calculated ratios between capillary vs. vdW and coupling force due to ice vs. capillary forces were analyzed for model verification. Both calculated ratios had similar behavior, with high values for small microobjects and decreasing values for larger microobjects. The coupling force due to ice was very large for smaller microobjects compared to for the capillary force; however, the ratios equalized for larger microobjects. Note that the coupling force due to ice in the theoretical model was calculated from the ice surface, which was the same as in the capillary calculations for the optimal meniscus conditions (the largest capillary force possible). Where, for larger microobjects, the capillary force becomes insufficient, the amount of water (or ice surface) between the microobjects can be increased further; hence, the coupling force due to ice also increases, to the enable manipulation of larger submillimeter objects. In this manner, the meniscus is reshaped from a concave to a convex shape, with more surface area for ice coupling (as can be seen in Videos S11 and S12, showing the manipulation of submillimeter-sized objects).

### 3.5. Methods

To measure the pull-off forces between the microobjects, we used a force measurement method with a flexible traverse. The results were later analyzed and compared with the model outputs. For the manipulation of microobjects with the one-finger gripper, we used the previously developed vdW and capillary force methods and present a method with coupling force due to ice, yielding a universal method with which all three methods can be used in the same set-up for the gripping/release of microobjects.

#### 3.5.1. Pull-Off Force Measurement Method for Microobjects

**Figure 5:**
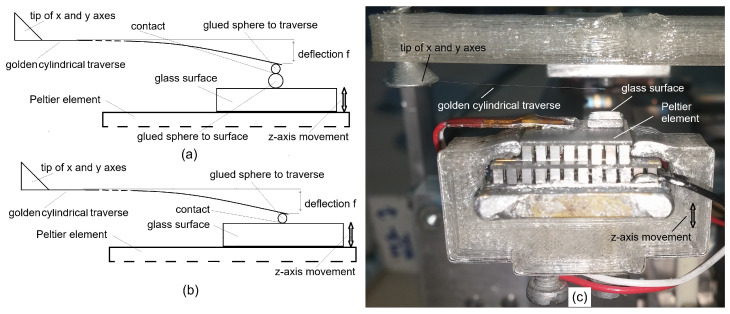
(**a**) Schematic of a pull-off force measurement between two spheres. (**b**) Schematic of a pull-off force measurement between a sphere and a surface. (**c**) Lab testbed for a pull-off method. Remark: The schematics are not drawn to scale.

The pull-off force *F* attracts microobjects when they come into contact. In our case, these forces can be vdW when the contact is dry, capillary forces when the contact is wet, or forces of mechanical coupling due to ice between the objects. When the lower object is pulled down by force *F* along the *z*-axis of the nanoprecision robot (see Equation (11), Figure 5a,b), the traverse is deflected with distance *f* towards the *z*-axis. The objects are “stuck” together during deflection due to the attraction force, and the microobjects (see Figure 3a) or the microobject and glass surface (see Figure 5b) are stuck together until the opposite traverse elastic force becomes equal to the attraction force. When the elastic force becomes greater than the attraction force, the traverse with the microobject glued on the tip of the traverse tears away from the lower object towards a position of equilibrium and the deflection *f* is read at that moment. The elastic golden traverse is firmly mounted: with one side mounted onto the tip of the x-y axes of the nanoprecision robot system (see Figure 5c), and the other side with the glued microobject left free. The Peltier element of the *z*-axis is cooled or heated to produce a dry contact between the microobjects (a temperature above the dew point temperature), a thin layer of condensed water in the contact (a temperature below the dew point temperature but above the freezing point temperature), or a thin layer of ice around the contact. The equation for calculating the pull-off force *F* of the traverse with a circular cross-section is given by Equation (11):

(11) $$F=\frac{3\cdot E\cdot f\cdot \pi \cdot d^{4}}{64\cdot l_{T}^{3}},$$

where the length of the golden traverse is $l_{T}=20$ mm, the diameter of the golden traverse is *d* = 50 µm, Young’s modulus of gold is E=79 GPa, and *f* is the deflection of the golden traverse (typically a few hundred µm). A more precise description of the pull-off measurement method is presented in [14,24,32]. The precision of the method is approx. ±25 nN, if *f* is accurate to ±61 nm, as it was in our case.

#### 3.5.2. Movement of Microobjects with a One-Finger Microgripper

Our new one-finger gripper uses the same equipment as our previously developed methods, described individually in [17,24]. It operates within a pressure chamber with a controlled atmosphere of RH = 30%, p=1 bar. We only had to modify the humidity controller of the existing equipment. We use relatively dry atmospheric air with RH = 30% to obtain a very thin layer of water on the tip of the one-finger gripper to produce the capillary force, or a thin layer of ice on the tip of the one-finger gripper to freeze the object on the tip, and evaporate the water on the surface to prevent parasite capillary force during gripping. Another reason to use a thin layer of water or ice is that the time needed to condense and later freeze the thin layer of water is short, approx. 2–3 s.

Conversely, we melt the ice and evaporate the condensed water on the tip of the one-finger to prevent the existence of parasite capillary force between the object and tip of the one-finger gripper, and at the same time, we condense the thin layer of water when we want to produce capillary force between the object and surface, or a thin layer of ice on the surface to freeze the object on the surface to release the object. In this manner, we can produce and use the three types of forces in each contact between the object and the tip of the one-finger gripper or between the object and surface: vdW, when the contacts are dry; capillary force, when the contacts have a thin layer of condensed water; and coupled force due to the ice around contacts.

This is interesting because vdW is the weakest force (range: nN–μN), the capillary force is about 5–9 times higher, and the coupled force due to ice is approx. 11–13 times higher than the vdW for same-sized objects (see Section 3.1). We can produce all possible combinations of these three forces in both the tip–object and object—surface contacts. Therefore, a single device that can grip objects from a surface and release them onto a surface can be extended to objects with dimensions from a submicrometer to submillimeter area. Also, another important advantage is that a single device that can leverage three different forces is much more reliable for gripping/releasing tasks than one with only one or two forces. This is because parasitic capillary forces tend to arise due to the temperature, pressure, and moisture of the ambient air in the microvicinity of the microobject, which is very difficult to control. Therefore, for example, if the object cannot be released by the capillary force between the microobject and glass surface, due to the parasitic capillary force between the tip of the finger and microobject, then we just simply decrease the temperature of the surface below the freezing temperature, and thus the force between the microobject and surface is coupling force due to ice and release is possible. Conversely, if a parasitic capillary force exists between the microobject and glass surface, then it is impossible to grip the microobject with capillary force, but it is possible to decrease the temperature of the one-finger gripper below the freezing temperature, and thus gripping, as described previously, is successful.

Our method uses coupling force due to ice for gripping and gravitational force for releasing only for objects with submillimeter sizes, while for microobjects with dimensions below 100 µm, the vdW, capillary force, and coupling force due to ice are used, because the gravitational force is much smaller.

(a) Capillary–van der Waals forces: procedure for microobjects

**Figure 6:**
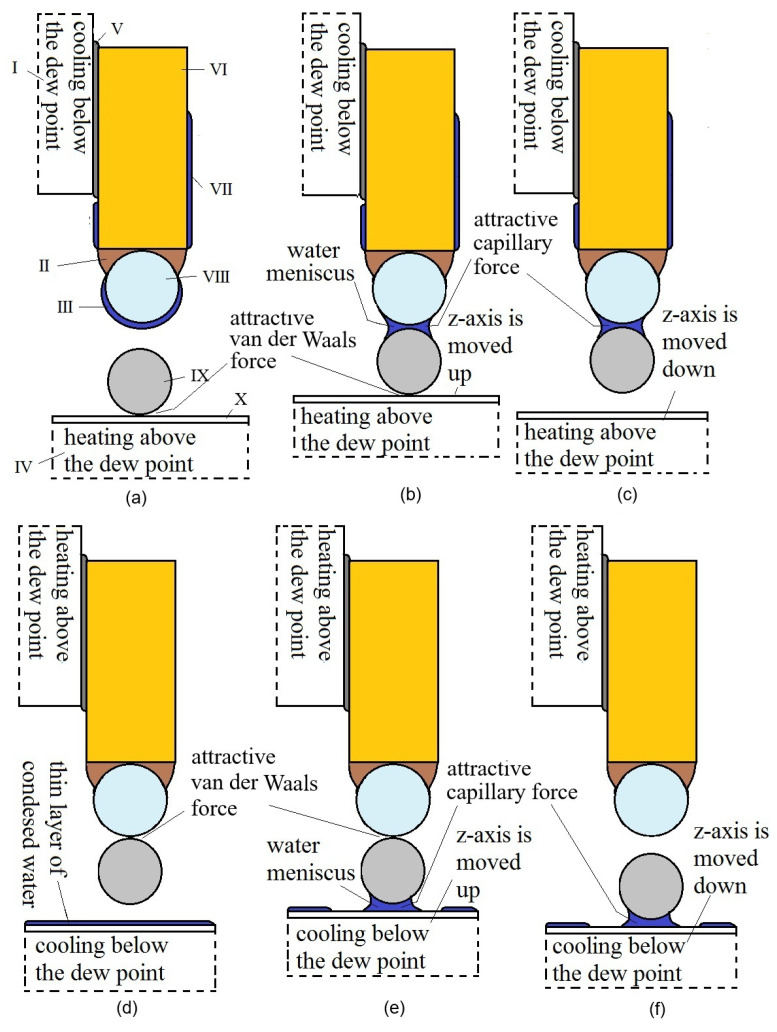
Gripping and releasing procedures for capillary–van der Waals forces (I—Peltier element *x*-axis; II—hydrophobic epoxy adhesive; III—condensed layer of water; IV—Peltier element *z*-axis; V—heat-conducting paste; VI—golden wire with diameter *d* = 50 µm; VII—thin layer of condensed water; VIII—a tip of the one-finger gripper; IX—the spherical microobject, with *d* = 5–60 µm). (**a**) The one-finger gripper is approached to overhead of the microobject. (**b**) The one-finger gripper touches with the microobject, water meniscus is formed, capillary force starts to interact. (**c**) The one-finger gripper lifts the microobject. (**d**) The water meniscus is removed by heating the one-finger gripper. (**e**) The microobject touches with the plane, water meniscus is formed between the two, capillary force starts to interact. (**f**) The one-finger gripper is distanced from the microobject, leaving the microobject on the plane.

Figure 6 shows the gripping and release of a microobject if we use only vdW and capillary forces. This procedure is similar to that of our own method published in [17]. By comparison, our newly developed procedure is more reliable, due to the constant RH in the chamber. The first drawback of our previous method [17] is that the temperature of the dew point varies with the humidity of the air in the chamber, requiring a long initial procedure to determine the temperature of the dew point. The second drawback is the prolonged time required for releasing/gripping. If the temperature of the dew point varies due to fluctuation in the atmospheric RH during repeated gripping/releasing tasks over a period of a few minutes, then the amount of condensed water on the one-finger gripper is not reliable. If there is too little condensed water, gripping is not possible. If there is too much, the time to grip or release the microobject can be up to 10 times longer than with our improved method due to faster condensation and longer evaporation. We have observed that 28% < RH < 33% is the best composition, and consequently, the gripping/releasing procedure lasts approx. 2–3 s. This kind of reliability is important if we wish to automate the gripping/releasing procedure.

The capillary–vdW procedure for gripping and releasing a microobject is as follows (see Figure 6):

- The temperature of the one-finger gripper is decreased below that of the dew point by about 1 to 2 °C. The thin layer of water is condensed on the tip of the one-finger gripper. We could not measure the depth of the condensed water because it was seen on the top of the one-finger gripper only through reflections off the tip (at low intensities) due to condensed dew. At the same time, the object positioned on the surface plane has to be heated above the temperature of the dew point by 1 to 2 °C, so the parasitic capillary force disappears due to the evaporation of dew between the object and surface, if it exists. The thin layer of water that condensed in the golden trunk of the one-finger gripper cannot move through the hydrophobic epoxy adhesive due to its hydrophobic property (see Figure 6a).
- The *z*-axis with the microobject moves up and makes slight contact with the tip of the one-finger gripper. The thin layer of water on the tip produces a water meniscus between the microobject and the tip (see Figure 6b).
- The attractive capillary force between the microobject and the tip of a one-finger gripper is greater than the vdW between the microobject and surface plane, so when the *z*-axis is moved down, the microobject remains in contact with the tip of the one-finger gripper (see Figure 6c).
- The release procedure starts with increasing the temperature of the tip of the one-finger gripper above the dew point temperature by 1 to 2 °C, so all condensed water between the tip and microobject evaporates, and the parasitic capillary force is consequently eliminated. Only an attractive vdW exists between the microobject and tip. At the same time, the surface plane temperature is decreased below the dew point temperature, and a thin layer of water condenses on the surface (see Figure 6d).
- The *z*-axis is moved up. The condensed layer of water produces a meniscus between the microobject and surface, and consequently, the capillary force is created between them (see Figure 6e).
- The *z*-axis is moved down, and because the capillary force between the microobject and surface is greater than the vdW between the microobject and tip, the microobject attaches to the surface.

Note that the temperatures above or below the dew point temperature must only differ by 1 to 2 °C. Higher temperature ranges would produce parasitic capillary forces instead of vdW and, consequently, would not have be useful for reliable gripping/release procedures.

(b) Coupling force due to ice–capillary force: procedure for microobjects

**Figure 7:**
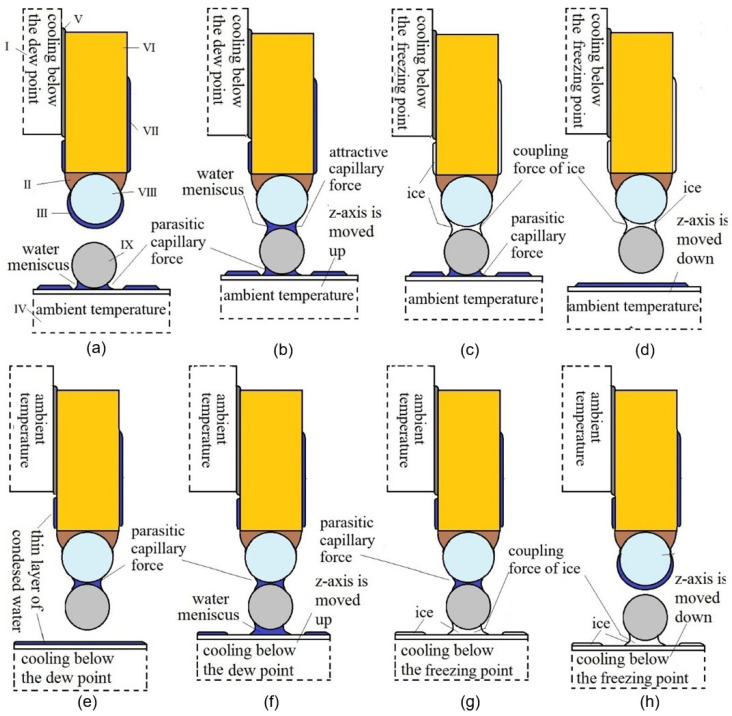
Coupling force due to ice–capillary force procedure (I—Peltier element *x*-axis; II—hydrophobic epoxy adhesive; III—condensed layer of water; IV—Peltier element *z*-axis; V—heat-conducting paste; VI—golden wire with diameter *d* = 50 µm; VII—thin layer of condensed water; VIII—a tip of the one-finger gripper; IX—a spherical microobject, with *d* = 5–60 µm). (**a**) The one-finger gripper is approached to overhead of the microobject. (**b**) The one-finger gripper touches with the microobject, water meniscus arises, capillary force starts to interact. (**c**) Ice forms between the one-finger gripper and the microobject. (**d**) The one-finger gripper lifts the microobject. (**e**) The ice is removed by heating the one-finger gripper. (**f**) The microobject touches with the plane, water meniscus forms between the two, capillary force starts to interact. (**g**) Ice forms between the plane and the microobject by cooling the plane. (**h**) The one-finger gripper is distanced from the microobject, leaving the microobject on the bed.

If the ambient temperature of the air is below the dew point temperature and above the freezing point of water, then the coupling force due to ice–capillary force procedure for gripping and releasing a microobject is as follows (see Figure 7):

- Despite the ambient temperature being below the dew point, we must set the temperature of the tip of the one-finger gripper above the dew point temperature, so an unknown quantity of water on the tip of the one-finger gripper evaporates. Later, we must decrease the temperature of the tip of the one-finger gripper below the dew point temperature by 1 to 2 °C for 1 s, so only a thin layer of water condenses on the tip of the one-finger gripper. Between the microobject and plane surface, there is a thin layer of water, so an attractive parasite capillary force exists between them (see Figure 7a).
- The *z*-axis is moved up, and a water meniscus is created between the tip of the one-finger gripper and the microobject. The attractive capillary force crated between the microobject and tip is usually not enough to overcome the parasitic capillary force between the microobject and surface (see Figure 7b).
- The temperature of the tip is cooled below the freezing point of water by 1 to 2 °C for another second, so the water meniscus between the microobject and tip is frozen, and consequently, the coupling force of ice is created between the microobject and the tip of the one-finger gripper (see Figure 7c).
- The coupling force due to ice between the microobject and tip is greater than the capillary force; thus, when the *z*-axis is moved down, the microobject remains attached to the tip of the one-finger gripper (see Figure 7d).
- The release part of the procedure starts with the heating of the plane surface above the dew point temperature to evaporate an unknown amount of water on the surface. The surface is then cooled below the temperature of the dew point by 1 to 2 °C for 1 s, so that the thin layer of water condenses on the surface of the plane. At the same time, the ambient temperature produces a water meniscus between the microobject and the tip of the one-finger gripper and, consequently, the parasitic capillary force between them (see Figure 7e).
- The *z*-axis is moved up, and another meniscus is created between the surface of the plane and the microobject. The capillary force between the plane’s surface and microobject is usually not enough to attach the object to the tip of a one-finger gripper (see Figure 7f).
- The surface plane is cooled below the freezing temperature of the water by 1 to 2 °C, so the meniscus of water between the microobject and the surface of the plane is frozen. This ice produces the coupling force due to ice between the microobject and plane surface, which is greater than the parasitic capillary force between the tip of the one-finger gripper and the microobject (see Figure 7g).
- The *z*-axis is moved down with the attached microobject (see Figure 7h).

Note that if the ambient temperature is above the temperature of the dew point, then the gripping/releasing procedure is similar to that described in Figure 3. However, it is not reliable, due to the unknown temperature above the dew point, which may disturb the creation of the water meniscus and, consequently, the attraction of a capillary force between the tip of the one-finger gripper microobject during gripping, or the attraction of the capillary force between the microobject and the plane surface during release.

## 4. Results

The experiments were performed in a laboratory nanorobotic chamber under ambient room conditions with controlled RH. For demonstration purposes, the experiments were performed with spherical objects and objects of irregular shapes. Initially, the pull-off force was measured using a gold traverse. Then, gripping and releasing experiments were performed with the aforementioned objects. The experiments were performed with the aim of demonstrating the following:

- The use of three different forces (vdW, capillary force, coupling force due to ice) with a single one-finger gripper;
- Reliable and fast gripping and release of microobjects and submillimeter-sized objects,
- Universal application to microobjects and submillimeter-sized spheres, as well as objects of irregular geometry.

The experiments are detailed in the following section.

### 4.1. Pull-Off Force Measurements

**Figure 8:**
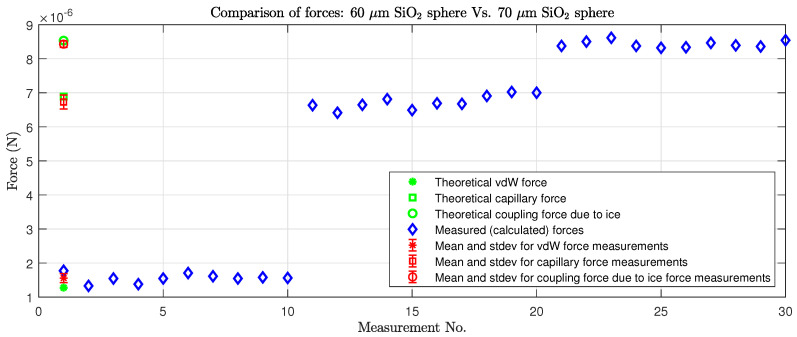
Measurements of forces between microobjects: a 60 µm sphere and a plane.

**Figure 9:**
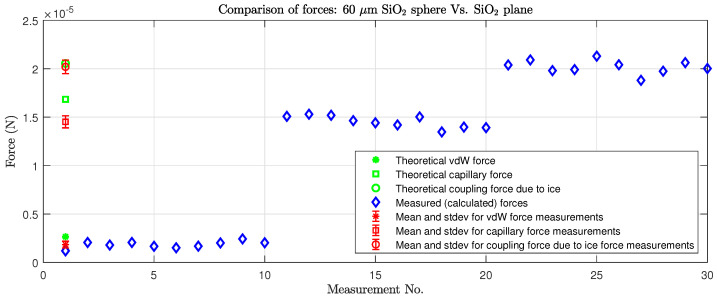
Measurements of forces between two objects and a plane.

We measured the vdW, capillary forces, and coupling force due to ice between microobjects for two different combinations of objects (number of measurements N=10), using the measurement method described in Section 3.5.1. During the measurements, the reference ambient conditions were set at RH = 30%, T = 22 °C, and approx. atmospheric pressure *p* = 1 bar. The following pull-off force measurements were taken to evaluate the strength of each force statistically, and the values were compared with the theoretically calculated Equations (1)–(4):

- Between two spherical objects with diameters 60 µm and 70 µm (see Figure 8);
- Between a spherical microobject with diameter 60 µm and a plane (see Figure 9).

Figure 8 and Figure 9 show the individual measurements, from which the arithmetic mean (or average) and standard deviation (stdev) of each type of measurement can be calculated (Table 4).

Regarding practical conditions such as sensitivity conditions, we note that they notably affect the dynamics of the water/ice meniscus formation and evaporation. In particular, the RH value should be set appropriately to ensure that the water meniscus forms from the atmosphere water in the vicinity of the cooled tip; however, if the formation is too fast, it is difficult to control the optimal size and shape to produce an adequate capillary force. The discovery of a dew/freezing point at the end of the tip is connected to thermal losses due to the long tip from the Peltier element to the end of the tip. Therefore, before each microobject manipulation, these points must be obtained prior to operation with a quick test (tip cooling, water evaporation). Active release of the microobject is the most challenging step; however, when using a combination of capillary force for gripping/releasing vs. vdW on the opposite side, most regular small- and medium-sized microobjects can be manipulated reliably. When it comes to larger or irregularly shaped objects, the most reliable gripping procedure relies on coupling force due to ice, and releasing on capillary force on the opposite side. The release becomes easy as the ice on the top of the gripper evaporates and the dominating force on top becomes vdW vs. capillary force on the bottom, which is larger by definition.

### 4.2. Experiments with the One-Finger Gripper

Experiments were performed with the same one-finger gripper for spherical microobjects and submillimeter-sized objects with irregular geometry; a SiO_2_ sphere with a diameter of *d* = 60 µm was glued onto the tip of a golden wire with a diameter of *d* = 50 µm and length of 5 mm over the lower edge of the Peltier element mounted on the tip of the x−y axes (see Figure 2b). The experiments aimed to prove that the same gripper set-up can be used to grip and release microobjects to submillimeter-sized objects of spherical to irregular geometric shapes with three forces: vdW, capillary force, and coupling force due to ice. All experiments were carried out at an atmospheric pressure of 1 bar and RH ≈ 30%. The temperature of the dew point was between 6 and 7 °C. All experiments were recorded, and are provided as videos in the Supplementary Material. The set-up temperatures were set in the range of −5 °C to −25 °C for ice formation and +5 °C to +15 °C for dew evaporation.

Video S1 presents a single measurement of the vdW between the spherical object and a plane, while Video S2 presents a single measurement of the vdW between two spherical objects. The distance *d* between the microobjects in contact was determined using the force measurement method described by Bratina et al. [26], where the contact distance for the sphere-plane combination was slightly smaller than for the sphere-sphere combination. In the first case, with the plane glass as a microscope slide and the sphere as a glass bead, the contact was determined at d=0.37 nm. In the second case, both spheres were from the same batch, and the contact distance was determined at d=0.42 nm. Hence, the microscope glass appears to be smoother than the surface of the glass beads. These values were also used for the theoretical calculations of the forces.

#### 4.2.1. Experiments on Spherical Microobject Manipulation with the One-Finger Gripper

- Experiments with vdW and coupling force due to ice: All three experiments use the coupling force due to ice as a gripping force between the tip of the one-finger gripper and spherical object (contact temperature of −5 to −25 °C), and the vdW as the opposite force between the object and surface (contact temperature of 10 to 20 °C) during gripping. Releasing is performed with the coupling force due to ice between the surface and object (contact temperature of −5 to − 25°C) and vdW between the object and the tip of the one-finger gripper (contact temperature of 10 to 20 °C). Video S3 presents the movement of a spherical object with diameter *d* = 25 µm, Video S4 presents the movement of the spherical object with diameter *d* = 60 µm, and Video S5 presents the movement of the spherical object with diameter *d* = 100 µm.
- Experiments with a capillary force and coupling force due to ice: The experiment uses the coupling force due to ice as a gripping force between the tip of the one-finger gripper and the spherical object (contact temperature of −25 °C) and the capillary force as the opposite force between the object and surface (contact temperature of 5 °C) during gripping. Releasing is performed with the coupling force due to ice between the surface and object (contact temperature of −9 °C) and the capillary force between the object and the tip of the one-finger gripper (contact temperature of 5 °C). Video S6 presents the movement of the spherical object with a diameter greater than *d* = 60 µm.
- Experiments with capillary force and vdW: The experiment uses the movement resulting from the capillary force between the tip of the one-finger gripper and the spherical object (contact temperature of 5 °C), where vdW is the opposite force between the object and surface (contact temperature of 15 °C) during gripping. Releasing is performed with the capillary force between the surface and object (contact temperature of 5 °C), and the vdW between the object and the tip of the one-finger gripper (contact temperature of 5 °C). Video S7 presents the movement of the spherical object with the diameter described above *d* = 25 µm.
- Experiments with combinations of forces: Video S8 shows the experiment in which gripping is performed with the coupling force due to ice between the tip of the one-finger gripper and the capillary force between the object with diameter *d* = 60 µm and the gripper, while the release is performed with the coupling force due to ice between the surface and object and the vdW between the object and finger. Video S9 shows the experiment in which gripping is performed with coupling force due to ice between the tip of the one-finger gripper and the vdW between the object with diameter *d* = 50 µm and the gripper, while the release is performed with coupling force due to ice between the surface and object and capillary force between the object and finger.
- Experiment with the smallest object: Video S10 shows the movement of the smallest spherical object with diameter *d* = 5 µm.

#### 4.2.2. Manipulation Experiments on Microobjects and Submillimeter-Sized Objects with Irregular Geometry Using the One-Finger Gripper

Video S11 shows the gripping and release of a microobject with irregular geometry and a dimension of approx. 100 µm. Video S12 shows the same movement with an object with an irregular geometry of approx. 170 µm. Video S13 again shows the gripping and release of an object with an irregular geometry of approx. 300 µm. This was the largest object with which active release was possible. Finally, Video S14, shows the successful gripping of an object with an irregular geometry of approx. 350 µm, where gripping was successful, but release was possible only with gravitational force. If we increased the temperature above the freezing point, the capillary force or vdW was not enough to overcome the gravitational force of the object if it simply fell from the gripper. The objects were small particles of silica gel that were crushed down to the desired dimensions.

## 5. Discussion

### 5.1. Experiments on Pull-Off Force Measurements

According to Section 3.5.1 and Section 4.1, the coupling force due to ice is the most powerful attracting force, followed by capillary force and then vdW as the weakest (see also Table 2 and Table 3). Table 2 and Table 3 show the theoretically calculated capillary force and vdW for spheres of different diameters (5 µm < d < 100 µm) regarding contact between two spheres and between a plane and a sphere (see Equations (1)–(6). The data show that the ratio between the capillary force and vdW for microobjects varies between 6.15 and 10.74, depending on the sphere–sphere or sphere–plane combination. The ratio between the theoretically calculated vdW and coupling force due to ice was even higher (from 1 to 20.47) and is comparable for gripping and releasing procedures. The later ratios were determined for an optimal meniscus shape of water/ice and its surface for the creation of a coupling force due to ice. If even larger forces are required, the meniscus can be reshaped from a concave to a convex shape by increasing the amount of water between the microobjects in contact to cover more of the surface, hence producing a larger coupling force due to ice. The experimental results of the pull-off force measurements are shown in Table 4, N=10. Although the measured values are close to the theoretical calculations, some deviations were identified. Force equations and models rely on data and coefficients such as the Hamaker coefficient, which is difficult to obtain for some materials. We used the values found in most related works and the literature. Therefore, the differences in the compared values are understandable and expected. The measurement conditions also have an effect on the measured force; the traverse can be difficult to define (regarding length, diameter, purity, Young’s coefficient, etc.), and we rely on the production specifications of materials. In addition, ambient conditions can cause issues, where instead of pure dry contact between microobjects, a small capillary force can arise, affecting the force measurement, or, due to deformation, the object’s surface breaks the force in contact between the objects. However, with the derived models and calculated forces and ratios, the values are in accordance with expectations and confirmed by the measurements.

Table 5 summarizes the key indicators and evaluations of the success rate for each acting force.

### 5.2. Gripping and Release of Spherical and Irregular Geometry Objects

All three mentioned forces (vdW, capillary force, and coupling force due to ice) can be used for gripping and releasing procedures with a one-finger gripper for microobjects with sizes smaller than 100 µm, where the microobject is spherical or has irregular geometry. The presented one-finger gripper with supporting devices (temperature controllers with Peltier elements, x−y−z Cartesian nanoprecision robot mechanism, humidity controller, and others (see Section 2.2) provides us with an opportunity to use all three forces to achieve the most suitable conditions for the reliable gripping and release of microobjects. The experiments (see Section 4.2) showed that the objects can be even in the submillimeter range, up to 0.3 mm. A larger object, with a size above 0.4 mm, has a larger gravitational force than the capillary force or the coupling force due to ice. The smallest object specified in the experiments was a sphere with a diameter of 5 µm. Thus, the range of objects with irregular geometry or spherical objects can be between 5 µm and 300 µm, where the tip of the one-finger gripper has a diameter of 60 µm, as presented in our experiments. If we decrease the diameter of the sphere attached to the tip of the golden wire to 30 µm, then it is possible to move an object with a diameter of 2 µm; however, the upper limits of the object’s range will decrease to approx. 150 µm, and conversely, if the diameter of the sphere on the tip of the one-finger gripper increases, the lower and upper limit of the object’s range also increases. We believe that the longest range and most reliable gripping and release with all three forces (vdW, capillary force in coupling force due to ice) is possible when the diameter of the sphere glued to the tip of the gripper of one-finger is approx. 60 µm.

Regarding the RH, we discovered that RH ≈ 30% is the most suitable to ensure reliable and fast movement of objects in a range between 5 µm and 300 µm. RH > 30% produces too much water in the contact area in the case of attractive capillary force, so the capillary force is weaker, or even disappears completely. In the case of the coupling force due to ice when RH > 30%, a lot of ice is created, requiring a lot of time to melt it. The melting time could be up to 30 s long, while, when the air RH = 30%, the melting time of the accumulated ice is 2–3 s. If the air is of RH < 30%, then it takes longer to accumulate the optimum amount of water or ice for the capillary force or the coupling force due to ice in the contacts. We believe that RH = 30% is optimal, and it takes 2–3 s to accumulate enough water in the contact area to produce a reliable capillary force, and another 3 s for the water to freeze to achieve a reliable coupling force due to ice if necessary. In the case of RH = 30%, the temperature of the dew point was approx. 5–7 °C if the temperature of the ambient air in the chamber ranged between 18 and 21 °C. Higher air temperatures in the chamber could lead to more time required for gripping or releasing. This is also true for larger objects. Our experiments show that if the object is larger than 300 µm, then the time to produce a sufficiently high coupling force due to ice can be extended by several seconds to up to 10 s.

The human operator of the gripping and releasing equipment must be trained to manipulate microobjects and submillimeter-sized objects. The most important skill is that, before the capillary force or coupling force due to ice is created, precise contact must be made between the object and surface, or between the object and the tip of the one-finger-gripper. The operator must also know whether the capillary force or coupling force due to ice is strong enough to grip the object. When creating capillary force, the operator must wait until a thin layer of dew accumulates on the surface of the tip and the object. When creating coupling force due to ice, the operator must wait until the accumulated dew starts to crystallize (freeze) on the tip of the one-finger gripper and on the object. Crystallization ends when the object stops turning restlessly; it is then the right time to grip or release the object (see the experiments in Section 4.2, Video S5).

The time to change the temperature is crucial for fast gripping and releasing procedures. It can be reduced with more powerful Peltier elements and a temperature controller with a faster settling time, as well as by reducing the length of golden wire used as the heat transfer conductor. In our case, the length of the golden wire that exceeds the edge of the Peltier element was approx. 5 mm. The length cannot be any shorter due to the geometrical shape of the Peltier element and surrounding devices, but it is possible to change this geometry in future developments of the nanoprecision robot mechanism.

The reliability of gripping/releasing with capillary force and vdW is greater than 90% for a microobject when the object shape is of primitive form (sphere, plane). For larger round objects in the submillimeter range down to the 200 µm range, the reliability is about 85%. For irregularly shaped microobjects and submillimeter-sized objects, the reliability with capillary force decreased to below 20%. This is because an appropriately shaped water meniscus cannot form between microobjects. Therefore, instead of capillary force, we can use coupling force due to ice, with which the reliability for an irregular shape was approx. 80%, while for a round object, it was only 60% for both microobjects and submillimeter-sized objects. For microobjects larger than 300 µm, the coupling force due to ice is no longer reliable (only about 10%). This is due to many factors: the effect of gravity (the objects become heavier); the tip being small according to the manipulated object (small contact with ice coupling surface); the temperature of larger microobjects having higher internal specific temperatures, making them difficult to cool down enough to obtain the required degree of ice coupling; and our inability to measure the temperature directly on the tip of the gripper, instead measuring from cca. 10 mm away from the Peltier element, resulting in poor measurements for the settling of dew points or freezing point temperatures and, consequently, in failure to grip or release the object. There is also the problem of the surrounding air temperatures and RH in the microvicinity of the gripper and objects, which were measured with sensors positioned a few cm away, leading to failures in gripping and releasing. Therefore, the proposed method is still not mature enough to achieve the fully automated grasping and release of microobjects, but it can be implemented for some circular microobjects when the capillary force and vdW are used for gripping and releasing procedures. Note that each irregularly shaped object requires its own procedure and thus a skilled human operator to perform it.

## Figures and Tables

**Table 2:** Theoretical force calculations for the three forces. The ratios are also calculated between the capillary force and vdW. SiO_2_ sphere 1 vs. SiO_2_ sphere 2.

Sphere 1: *d* = 60 µm	Sphere 2 Diameter			
	d = 5 µm	d = 25 µm	d = 60 µm	d = 100 µm
van der Waals force [µN]	0.14	0.54	0.92	1.15
Capillary force [µN]	1.52	4.54	6.56	7.56
Coupling force due to ice [µN]	31.10	13.10	9.01	7.63
Ratio (capillary/v.d. Waals)	10.74	8.37	7.12	6.56
Ratio (ice/capillary)	20.47	2.90	1.37	1.01

**Table 3:** Theoretical force calculations for the three forces. The ratios are also calculated between the capillary force and vdW. SiO_2_ sphere vs. SiO_2_ plane.

Plane	Sphere Diameter			
	d = 5 µm	d = 25 µm	d = 60 µm	d = 100 µm
van der Waals force [µN]	0.20	1.00	2.41	4.01
Capillary force [µN]	1.87	8.14	16.90	24.70
Coupling force due to ice [µN]	7.52	14.90	20.50	24.10
Ratio (capillary/v.d. Waals)	9.32	8.10	6.98	6.15
Ratio (ice/capillary)	4.01	1.83	1.22	0.97

**Table 4:** The experimental measurements of forces, as derived from Figure 8 and Figure 9. Operating conditions: RH = 30%, T = 22 °C, *p* = 1 bar. N=10. All forces are expressed in µN. * = coupling force due to ice.

Meas. No.	*sp-sp* Forces in µN			*sp-pl* Forces in µN		
	vdW	Capillary	Ice *	vdW	Capillary	Ice *
1	1.774	6.636	8.377	1.198	15.080	20.390
2	1.331	6.415	8.505	2.062	15.300	20.910
3	1.547	6.647	8.616	1.796	15.190	19.800
4	1.381	6.814	8.377	2.062	14.640	19.910
5	1.547	6.492	8.322	1.663	14.410	21.300
6	1.708	6.692	8.338	1.514	14.190	20.410
7	1.613	6.675	8.466	1.680	15.020	18.800
8	1.547	6.908	8.394	2.013	13.470	19.740
9	1.580	7.024	8.361	2.428	13.970	20.630
10	1.563	7.002	8.544	2.029	13.920	20.020
mean	1.559	6.731	8.430	1.845	14.520	20.190
median	1.555	6.684	8.386	1.904	14.526	20.203
stdev	0.132	0.204	0.098	0.348	0.626	0.700

**Table 5:** Summary table with regard to object sizes and success rates. Note: small objects, 5 µm–30 µm; medium objects, 30 µm–100 µm; large objects, 100 µm–300 µm. ice * = coupling force due to ice; cap ** = capillary force; SR *** = success rate.

	Min.	Typ.	Max.	Note
object size for cap * [µm]	5	60	100	small and medium spherical objects
object size for vdW [µm]	5	60	100	small and medium spherical objects
object size for ice ** [µm]	5	60	300	all objects, incl. objects of irregular shape
SR *** cap-vdW [%]		90		
SR *** ice-cap [%]		85		
RH [%]	28	30	33	defines freezing dynamics
Temp. range [°C]	20	22	24	ambient temperature

## Data Availability

The original contributions presented in this study are included in the article/Supplementary Material. Further inquiries can be directed to the corresponding author.

## Supplementary Materials

The following supporting information can be downloaded at: https://www.mdpi.com/article/10.3390/mi17050573/s1, Video S1: Measurement with traverse between sphere and plane, vdW force. Video S2: Measurement with traverse between two spheres, vdW force. Video S3: Gripping and releasing sphere, vdW and coupling force due to ice, *d* = 25 µm. Video S4: Gripping and releasing sphere, vdW and coupling force due to ice, *d* = 60 µm. Video S5: Gripping and releasing sphere, vdW and coupling force due to ice, *d* = 100 µm. Video S6: Gripping and releasing sphere, capillary force and coupling force due to ice. Video S7: Gripping and releasing sphere, capillary force and vdW. Video S8: Gripping capillary force and coupling force due to ice, releasing coupling force due to ice and vdW. Video S9: Gripping vdW and coupling force due to ice, releasing coupling force due to ice and capillary force. Video S10: Gripping vdW and coupling force due to ice, releasing coupling force due to ice and vdW, *d* = 5 µm. Video S11: Gripping and releasing irregularly shaped object, size of 100 µm. Video S12: Gripping and releasing irregularly shaped object, size of 170 µm. Video S13: Gripping and releasing irregularly shaped submillimeter-sized object, size of 300 µm. Video S14: Gripping irregularly shaped submillimeter-sized object, size of 400 µm.

## Abbreviations

The following abbreviations are used in this manuscript:

RH	Relative humidity (given in per cent)
vdW	van der Waals force
cap	capillary force
ice	coupling force due to ice
